# Poly-microbial *Clostridium cadaveris* bacteremia in an immune-compromised patient

**DOI:** 10.1093/omcr/omac146

**Published:** 2023-01-18

**Authors:** Jorge Abarca, Bassem Awada, Boris Itkin, Manyando Milupi

**Affiliations:** Division of Infectious Diseases, Department of Internal Medicine, Sultan Qaboos Comprehensive Cancer Care and Research Center, PO Box 566, Al Khoud, Muscat, Sultanate of Oman; Division of Infectious Diseases, Department of Internal Medicine, Sultan Qaboos Comprehensive Cancer Care and Research Center, PO Box 566, Al Khoud, Muscat, Sultanate of Oman; Division of Oncology, Department of Internal Medicine, Sultan Qaboos Comprehensive Cancer Care and Research Center, PO Box 566, Al Khoud, Muscat, Sultanate of Oman; Department of Laboratory Medicine, Sultan Qaboos Comprehensive Cancer Care and Research Center, PO Box 566, Al Khoud, Muscat, Sultanate of Oman

## Abstract

*Clostridium cadaveris* (*C. cadaveris*), a strict anaerobic gram-positive rod, is rarely reported in clinical specimens. Since its detection in 1899, it has always been linked to the decay of dead bodies. *C. cadaveris* is considered non-pathogenic to humans, however infrequently it can cause severe infections including bacteremia. The latter was typically associated with gastro-intestinal pathologies. We report the first case of *C. cadaveris* invasive infection at Sultanate Oman. The source was most probably an infected decubitus ulcer.

## INTRODUCTION

*Clostridium cadaveris* is a motile, slender-shaped, ubiquitous, anaerobic, gram-positive rod bacterium. It was first identified by Klein *et al*. in 1899, where it grew from rotting corpses [1]. In fact, *C. cadaveris* is one of the prominent bacteria that grow during the decay of dead bodies. It is considered one of the normal human gastro-intestinal (GI) flora, but it can occasionally cause severe infections.

Herein, we report a case of an elderly woman with a history of breast cancer on hormonal therapy and locally advanced left shoulder myxo-fibrosarcoma on local radiotherapy, who presents with septic shock caused by an infected decubitus ulcer. She was found to have poly-microbial bacteremia where blood cultures grew *C. cadaveris*, *Proteus mirabilis* and *Staphylococcus hominis*. The patient responded well to intravenous (IV) antimicrobial therapy and bedside debridement.

## CASE REPORT

**Figure 1:**
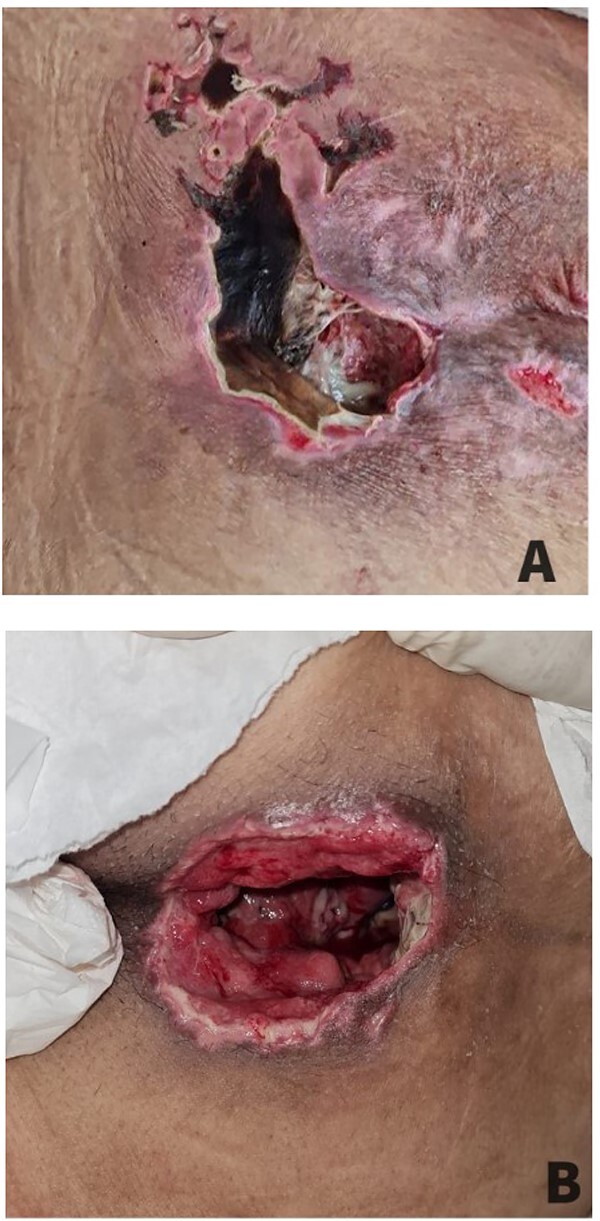
Infected sacral decubitus ulcer before and after debridement. (A) Infected sacral decubitus ulcer with necrotic tissue. (B) Infected decubitus post-debridement with healing tissue.

**Figure 2:**
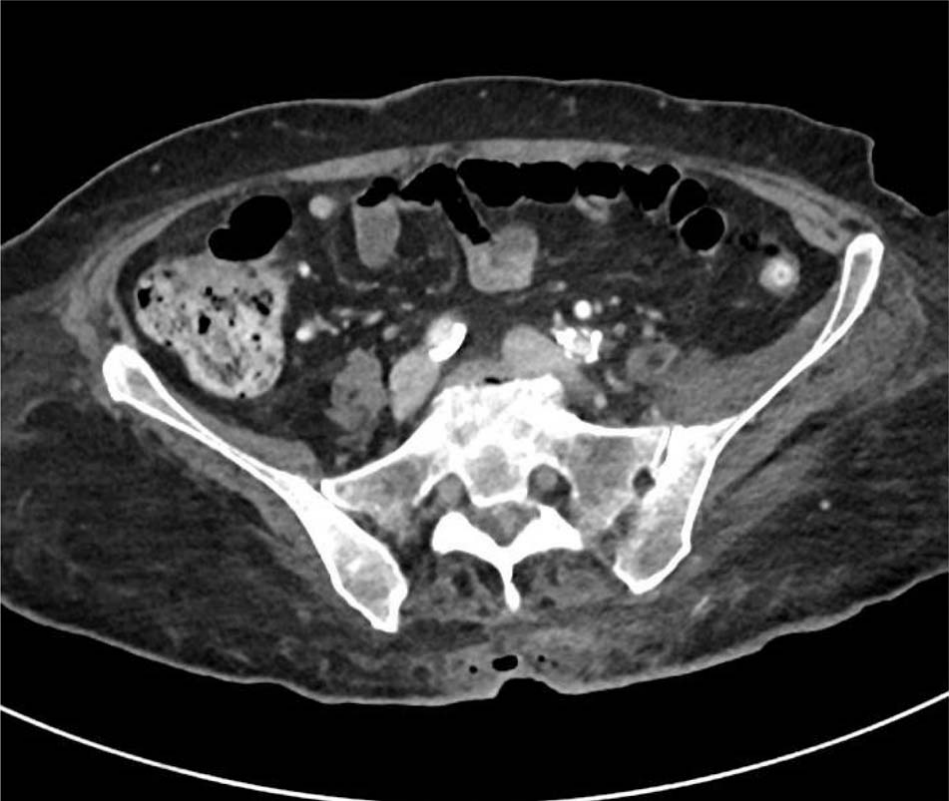
Coronal CT scan image of the pelvis showing fat stranding at the level of the sacrum with air locules.

A 72-year-old female patient with a history of hypertension, aortic stenosis post aortic valve replacement and dual malignancies of breast cancer on hormonal therapy and locally advanced shoulder myxo-fibrosarcoma on radiotherapy, presents to Sultan Qaboos University Hospital (SQUH) for hypotension and fever of one day’s duration. The physical examination was remarkable for an infected sacral decubitus ulcer; stage IV (Fig. 1A). Her labs on admission showed a white count of 19.3 x 10^9^ cells per liter with 92% neutrophils and 5% lymphocytes. Her C-reactive protein (C-RP) level was 207 mg/dl. She underwent a pelvis computed tomography (CT) with IV contrast, which showed fat stranding with air locules at the level of the sacral ulcer; however, it was negative for deep collections or osteomyelitis (Fig. 2). She was started empirically on meropenem 1 gram (gm) IV Q 8 hours plus vancomycin 1 gm IV Q 12 hours.

One set of blood cultures grew *C. cadaveris* and *S. hominis*, detected after 48 hours by using Matrix-assisted laser desorption/ionization-time of flight (MALDI-TOF) and the VITEK 2 automated system, respectively. The cut-off score of MALDI-TOF was 1.9, thus a repeat of speciation was done, that showed again *C. cadaveris* with the same score. The *C. cadaveris* isolate was susceptible to metronidazole with a minimum inhibitory concentration (MIC) of 0.032 detected by E test method. The isolate of *S. hominis* was susceptible to oxacillin with an MIC of 0.25. A deep swab culture from the decubitus ulcer grew *P. mirabilis*, which was susceptible to cefotaxime with an MIC < 1. She was, therefore, shifted to ceftriaxone 2 gm IV once daily plus metronidazole 500 mg IV Q 8 hours. The presence of prosthetic valve warranted the need to rule out the possibility of persistent bacteremia and endocarditis. For that, a third set of blood cultures and a TTE were done. The repeated blood culture grew *P. mirabilis*, with a similar susceptibility pattern as the previous isolate. The TTE result was unremarkable. Beside this, a bedside debridement and vacuum dressing was done by the wound care team. After 10 days of IV therapy, her white cell count dropped to 11 000 x 10^9^ cells per liter. Her C C-RP level decreased to 52 mg/dl. The patient was discharged on Amoxicillin-Clavulanate 1 gm bid for extra 1 week.

## DISCUSSION

Clostridium bacteremia contributes only 0.5–2% of total bacteremia [2]. It’s usually associated with GI pathologies. In fact, the disruption of the GI epithelium due to any pathology was found to be the main factor leading to Clostridium species bacteremia [3]. Furthermore, it is associated with a myriad of risk factors, including: malignancy, immune-suppression, poor physical condition, diabetes mellitus, decubitus ulcer, steroid therapy and chemotherapy [3]. In our case, the patient had a dual malignancy of breast cancer and myxo-fibrosarcoma, albeit she was not on an active chemotherapy regimen.

Although scarcely reported in the literature, *C. cadaveris* was found to be the causative microorganism to several severe infections including septic arthritis, lung empyema, peritonitis, splenic abscess, osteomyelitis and bacteremia [4–8]. There are only eight cases of *C. cadaveris* bacteremia in the literature (Table 1). The source of the infection in the reported cases was mostly the GI system. In our case, the potential source was the infected decubitus ulcer; especially that *P. mirabilis* grew on both wound and blood cultures. The inability to isolate *C. cadaveris* on the wound culture can be justified by the need for special handling when dealing with tissue or wound cultures. Also, the presence of enriched media in blood cultures may help in detecting such difficult to isolate micro-organism. To our knowledge, there is only one previous report with the same source of infection [3].

**Table 1:** Published case reports of *Clostridium cadaveris* bacteremia

Reference	Age–sex	Underlying comorbidities	Source of bacteremia	Antibiotic therapy	Prognosis
Schade *et al*. [9]	75–M	COPD	Acute diverticulitis	Amoxicillin, metronidazole and ciprofloxacin	Recovered
Schade *et al*. [9]	68–M	CVD	Probable gastrointestinal	Amoxicillin, metronidazole and ciprofloxacin	Death
Rasim Gucalp *et al*. [2]	61–F	RCC	Deep seated intestinal abscess	Vancomycin	Death, due to acute myocardial infarction
Rasim Gucalp *et al*. [2]	66–F	CLL with AIHA on prednisone 60 mg + IVIG DM type II, HTN	No clear source of infection	Metronidazole	Death, due to multiple comorbidities
Poduval RD *et al*. [3]	42–M	Paraplegia	Infected decubitus ulcer	Vancomycin plus rifampicin	Recovered
Morshed *et al*. [4]		Total hip arthroplasty, Metastatic Breast Cancer	Hip prosthetic arthritis	Clindamycin	Recovered
Xiangyun Li *et al*. [10]	74–F	Ovarian cancer	Intestinal source	Imipenem and cilasatin sodium	Recovered
Knight *et al*. [11]	19–M	Unremarkable	No clear source of infection	Metronidazole	Recovered

Abbreviations: COPD: chronic obstructive pulmonary disease; CLL: chronic lymphocytic leukemia; AIHA: autoimmune hemolytic anemia; IVIG: Intravenous Immunoglobulin; M: male; F: female; DM: Diabetes mellitus; HTN: hypertension; CVD: cardiovascular disease; RCC: renal cell carcinoma.

From the microbiology perspective, *C. cadaveris* can be identified by standard methods including API system, MALDI-TOF, Vitek 2 and 16 S rRNA sequencing [10, 12, 13]. MALDI-TOF is considered a proven useful method in detecting anaerobic organisms at the genus and species levels with a correct identification rate of at least 92% and 70%, respectively [12, 13]. For correct identification, the MALDI-TOF’s manufacturer recommended a cutoff score of 2, however Schmitt *et al*. showed that lowering the score value to 1.8 would increase the species-level identification from 70% to at least 85% [13]. In fact, other studies showed similar results, where cutoff score of 1.8 was recognized to increase the correct identification [14, 15]. *C. cadaveris* is usually susceptible to Metronidazole, which is considered the first line therapy [10]. Back to our patient, she received a total of 14 days of IV antimicrobial therapy in addition to bedside debridement, with a good response (figure 2). She was discharged on amoxicillin/clavulanate per os (PO) for an extra 1 week.

In summary, *C. cadaveris* bacteremia is extremely rare, where it mainly occurs in patients with GI pathology. Other main risk factors include malignancy, immune-suppression, chronic debilitating status, decubitus ulcers and diabetes mellitus. The report highlights the importance of anaerobic blood cultures and the role of MALDI-TOF in increasing the detection yield of the anaerobic bacteria.

## AUTHORS’ CONTRIBUTION

Dr Bassem Awada and Dr Jorge Abarca contributed equally to writing the manuscript, literature review and revision of the manuscript.

Dr Boris contributed to writing the manuscript.

Dr Manyando Mulipi contributed to writing the manuscript and data analysis.
